# Short-Term Oral Administration of 1.5 μg/kg bw/day of Deoxynivalenol Significantly Exacerbates Inflammatory and Itching Symptoms in a Mouse Model of Imiquimod-Induced Psoriasis

**DOI:** 10.3390/toxins17020047

**Published:** 2025-01-21

**Authors:** Takayoshi Miyamoto, Mariko Komuro, Ryota Aihara, Chiharu Ohira, Mao Kaneki, Naoki Iwashita, Yoshiichi Takagi, Atsushi Miyasaka, Masayo Kushiro, Shiro Miyake, Tomoki Fukuyama

**Affiliations:** 1 School of Veterinary Medicine, Azabu University, 1-17-71 Fuchinobe, Chuo-ku, Sagamihara-shi 252-5201, Kanagawa, Japan; 2 Bioalch Co., Ltd., 3-28-61 Honshuku-cho, Fuchu-shi 183-0032, Tokyo, Japan; 3 Japan SLC, Inc., 85 Ohara-cho, Chuo-Ku, Hamamatsu City 431-1103, Shizuoka, Japan; 4 Kyushu Okinawa Agricultural Research Center, National Agriculture and Food Research Organization (NARO), Suya 2421, Koshi 861-1192, Kumamoto, Japan; 5 Faculty of Agriculture and Forestry Management, Tohoku Professional University of Agriculture and Forestry, 1366, Tsunozawa, Shinjo 996-0052, Yamagata, Japan; 6 Institute of Food Research, NARO, 2-1-12 Kannondai, Tsukuba 305-8642, Ibaraki, Japan; kushirom@affrc.go.jp; 7 Department of Food and Life Science, Azabu University, 1-17-71 Fuchinobe, Chuo-ku, Sagamihara-shi 252-5201, Kanagawa, Japan; 8 Center for Human and Animal Symbiosis Science, Azabu University, 1-17-71 Fuchinobe, Chuo-ku, Sagamihara-shi 252-5201, Kanagawa, Japan

**Keywords:** deoxynivalenol, mycotoxin, psoriasis, IL-17, TNF-α

## Abstract

Deoxynivalenol (DON) is a mycotoxin commonly found worldwide and is implicated in various health effects. We recently demonstrated that subacute oral exposure to DON significantly exacerbates symptoms of type 2 helper T-cell-mediated allergic diseases in a model. We aim to investigate the role of oral DON exposure in type 17 helper T-cell-mediated immunoreactive diseases using a mouse psoriasis model. Psoriasis was induced by the dermal administration of 5% imiquimod in female BALB/c mice. A standard rodent diet was supplemented with DON to achieve a final concentration of 0.3 ppm (1.5 μg/kg bw/day), which was administered daily for 14 days. Skin thickness, scratching behavior, and transepidermal water loss (TEWL) were continuously measured during imiquimod administration. Mice exposed to DON exhibited significant increases in skin thickness, TEWL, and scratching behavior. Histological evaluations revealed aggravated hyperplasia, neutrophil infiltration, and inflammatory cell accumulation in the dermis. Furthermore, DON exposure significantly increased the number of CD4+ helper T cells and CD11c+ MHC class II+ dendritic cells in the auricular lymph nodes, along with elevated TNF-α and IL-17 levels in stimulated T cells. The gene expression of IL-17 in skin tissue was also significantly up-regulated in DON-treated mice. Collectively, these findings suggest that oral exposure to DON aggravates symptoms in a mouse psoriasis model.

## 1. Introduction

Deoxynivalenol (DON) is a type-B trichothecene mycotoxin produced by *Fusarium graminearum* and *F. culmorum*. It widely contaminates cereal grains, including wheat, barley, rye, maize, and oats, causing significant toxicological impacts on both human and animal health [1,2]. Acute symptoms of trichothecene ingestion in humans and animals include gastroenteritis, anorexia, nausea, and growth retardation [3]. Emerging evidence also indicates that high concentrations of DON can damage tissues, including the liver and kidneys, in animals [4]. However, given the current contamination risks, chronic exposure to low concentrations of DON should receive more attention than exposure to acute high doses of toxicity. However, only a limited number of reports regarding the low concentration of DON toxicity are available. Several previous reports, including those by Meky et al. [5] and Pierron et al. [6], demonstrated that oral exposure to low concentrations of DON abnormally enhances cytokine production by humans and with the significant induction of a specific antibody response during vaccination. We recently demonstrated the involvement of DON exposure in the development of type 2 helper T cell (Th2)-dependent allergic diseases, including allergic asthma and atopic dermatitis. Oral exposure to subchronic low concentrations (0.3 ppm, 1.5 μg/kg bw/day) of DON significantly aggravated allergic inflammation in mouse models and in vitro immune cells [7,8]. Our findings show that DON directly over-activates dendritic cells, leading to the significant enhancement of interleukin (IL)-6 and tumor necrosis factor (TNF)-α. These cytokines, secreted by dendritic cells and Langerhans cells, differentiate from monocytes and play a pivotal role in developing Th2-related allergic symptoms, including atopic dermatitis and asthma. The activation of dendritic cells is also critical as an initial step in other Th-type immune disorders, such as psoriasis [9]. Dendritic cell activation leads to IL-23 production, promoting Th17 and Th22 cell differentiation, which play a vital role in the development of psoriasis symptoms [10].

In this study, we aimed to investigate whether oral exposure to low concentrations of DON adversely affects Th17-related immune disorders using a mouse model of psoriasis.

## 2. Results

**Figure 1:**
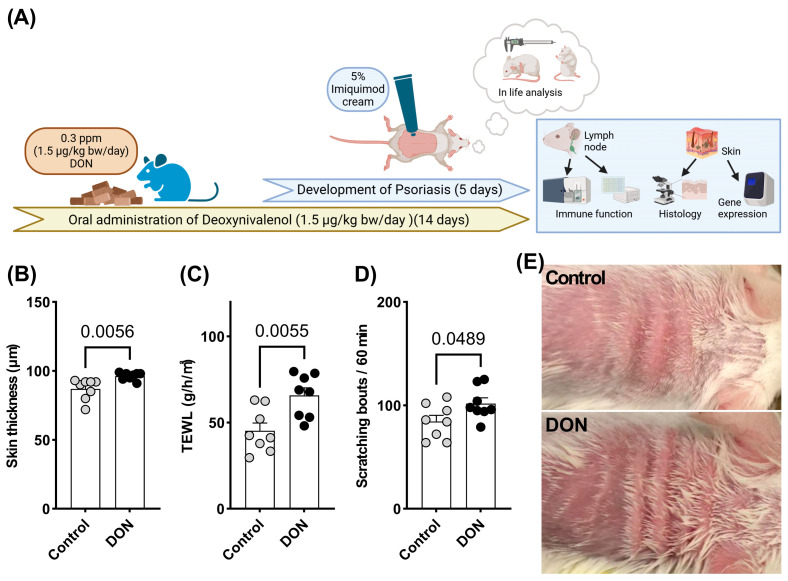
Experimental protocol for a mouse psoriasis model (**A**). Clinical symptoms of the mouse psoriasis model, including imiquimod-induced changes in back skin thickness (μm) (**B**), transepidermal water loss (TEWL, g/h/m^2^) (**C**), scratching bouts during 60 min (**D**), and typical images of back skin (**E**). The results are presented as the mean ± SEM (*n* = 8 per group). *p* < 0.05 (unpaired *t*-test) vs. the vehicle-only control group. DON = deoxynivalenol; TEWL = transepidermal water loss.

**Figure 2:**
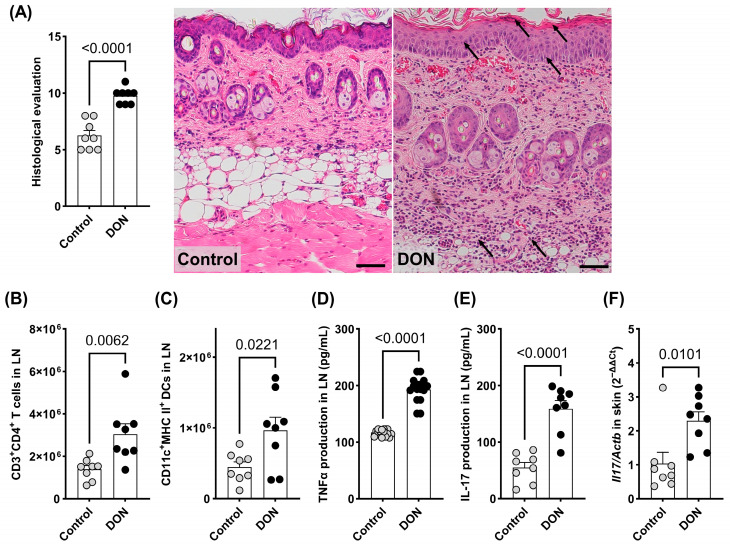
Total histological evaluation scores and typical histological images of back skin. Arrows indicate inflammatory cell infiltration in the epidermis and dermis for hyperplasia. (**A**). Effects of DON on immune functions, including the infiltration of CD3^+^CD4^+^ T cells (**B**) and CD11c^+^MHC class II^+^ dendritic cells (**C**) in auricular lymph nodes, and the secretion of TNF-α (**D**) and IL-17 (**E**) (pg/mL) by T cells. The gene expression of IL-17 in back skin samples was evaluated using the qPCR method (**F**). The results are presented as the mean ± SEM (*n* = 8 per group). *p* < 0.05 (unpaired *t*-test) vs. the imiquimod control group. LN = lymph node; TNF = tumor necrosis factor; IL = interleukin.

Oral exposure to DON for 14 days significantly increased back skin thickness (Figure 1B,E), TEWL (Figure 1C), and itching behavior (Figure 1D) compared to the imiquimod control group. The inflammatory response and breakdown of the cutaneous barrier induced by DON exposure were corroborated by a histological evaluation, which revealed that DON exposure significantly aggravates scores of inflammatory cell infiltration in the epidermis and dermis, as well as hyperplasia in the non-keratinized layer, compared to the imiquimod control group (Figure 2A, Table 1).

The influence of oral exposure to 1.5 μg/kg bw/day DON on immune function was examined by analyzing the number of immune cells and amount of cytokine secretion in auricular LNs. The number of helper T cells (CD3^+^CD4^+^, Figure 2B) and dendritic cells (CD11c^+^MHC class II^+^, Figure 2C) were significantly enhanced by DON exposure compared to the imiquimod control group. The significant increase in TNF-α and IL-17 production by CD3/CD28-stimulated T cells in the DON treatment group supports the enhanced activation of helper T cells and dendritic cells by DON (Figure 2D,E). Furthermore, oral exposure to DON significantly up-regulated IL-17 gene expression in back skin tissue compared to the imiquimod control group, indicating that type 3 innate lymphoid cells in the skin, as a source of IL-17, play an important role in the aggravation of psoriasis by DON exposure [11] (Figure 2F).

## 3. Discussion

In this study, we obtained new insights into the effects of low concentrations of DON present in food on the development of psoriasis. DON is a mycotoxin primarily found in cereals and grain-derived foods; however, its effects on the immune system and its role in inducing inflammation have not been extensively studied concerning its involvement in the pathophysiology of psoriasis. Psoriasis is a chronic inflammatory skin disease triggered by an autoimmune response primarily involving Th17 cells and characterized by excessive skin keratinization, inflammation, and itching [12]. This condition significantly affects the quality of life of patients. The imiquimod-induced mouse model, which also introduces strong Th17 and Th22 immune responses and is recognized as a typical model for human psoriasis, was used in this study to translate the mice data to humans [13]. Therefore, our findings suggest that even trace amounts of DON in food may contribute to the pathology of human psoriasis. The concentration of DON used in this study (0.3 ppm, 1.5 μg/kg bw/day) was selected based on our previous reports in a mouse model of atopic dermatitis and asthma [7,8]. The Recent Tolerable Daily Intake (TDI) of DON determined by the Food Safety Commission of Japan was 1 µg/kg bw/day, which is also a reason for dose selection [14]. The duration of exposure is also an important factor in evaluating the toxicity of chemicals. In this case, repeated exposure to low concentrations of DON (equivalent to the TDI level) was explored; therefore, the maximum duration of the exposure period of 14 days was selected based on the protocol to generate the psoriasis model.

Our results showed that DON exposure caused a significant increase in skin thickness and TEWL (Figure 1B,C), indicating impaired skin barrier function. An increase in scratching behavior, reflecting itching behavior (Figure 1D), was also observed. These results suggest that DON exacerbates skin inflammation. The significant enhancement of inflammatory cell infiltration observed in the histological evaluation (Table 1) supports this conclusion. As the current study focused on the early phase of psoriasis, TEWL, a barometer of the cutaneous barrier function, was not markedly increased by imiquimod application alone (the average value of TEWL in untreated mice was less than 10 based on our background data). However, compared to the vehicle treatment, DON exposure significantly enhanced TEWL, indicating that DON exposure promotes psoriasis symptoms at a subsequent stage. Furthermore, our findings showed that oral exposure to DON significantly enhanced itching behavior. Recent evidence suggests that IL-17A, released by commensal-specific Th17 cells upon injury, directly signals to sensory neurons via IL-17 receptor A, the expression of which is specifically up-regulated in injured neurons [15]. Th17 activation is closely correlated with the development of psoriasis and its associated itching behavior. Assessments of immune function showed that DON exposure significantly affected immune cell numbers and cytokine production in the LN and dorsal skin (Figure 2B–F). Specifically, the number of CD4^+^ helper T cells and CD11c^+^ MHC class II^+^ dendritic cells increased, suggesting the activation of the Th17 immune response. The production of TNF-α and IL-17 from CD3/CD28-stimulated T cells was also significantly increased, implicating these cytokines in the pathogenesis of psoriasis. Moreover, the increased gene expression of IL-17 in the back skin indicates that DON exposure contributes to psoriasis progression through Th17 cell activation. These findings suggest that DON plays a significant role in the pathological process of psoriasis and that strategies to eliminate DON or mitigate its effects could represent a novel approach to psoriasis treatment. Th17 cell activation also plays an important role in other immunomodulatory and neuromodulatory disorders, including asthma, atopic dermatitis, colitis, and Sjogren’s Syndrome [16,17]. Our previous and recent evidence demonstrated the strong relationship between DON exposure and the development of asthma, atopic dermatitis, and psoriasis [7,8], which means exposure to DON also influences other Th17-related disorders.

## 4. Conclusions

In conclusion, our study provides evidence that low concentrations of DON in food contribute to the pathogenesis of psoriasis through immune activation and skin inflammation. As we continue to unravel the complexities of this disease, understanding the role of environmental toxins such as DON is crucial for developing comprehensive treatment strategies that include both medical and lifestyle interventions. Addressing these factors may pave the way for improved patient outcomes and a better quality of life for those living with psoriasis. However, this study has limitations. Only 14 days of exposure to DON were conducted; therefore, actual chronic toxicological effects were not evaluated in this study. Additionally, the molecular mechanisms of DON that exacerbate inflammatory and itching symptoms should be examined for future studies.

## 5. Materials and Methods

### 5.1. Animals and Chemicals

Six-week-old female BALB/c mice were provided by Japan SLC, Inc. (Shizuoka, Japan). The mice were housed under a 12 h light/dark cycle at 22 ± 3 °C and humidity of 50% ± 20%. Food and water were provided ad libitum. All procedures in this study were conducted in accordance with the Animal Care and Use Program of Azabu University (Approval No. 1910097).

### 5.2. Oral Exposure to 1.5 μg/kg bw/day DON in a Mouse Psoriasis Model

A mouse psoriasis model was generated through the repetitive dermal application of 5% imiquimod cream (MOCHIDA PHARMACEUTICAL Co., Ltd., Tokyo, Japan), as shown in Figure 1A, according to Ando et al. [12]. A diet containing a low dose of DON (0.3 ppm, 1.5 μg/kg bw/day), prepared based on previous studies [5,6], was administered daily to the mice from 9 days before the initial application of imiquimod cream until the end of the experiment (Figure 1A). The actual concentration of DON in the diet (0.27+ ppm) was re-confirmed by ELISA (Elabscience^®^ Bionovation Inc., Houston, TX, USA). Back skin thickness (measured using a cutimeter, Mitutoyo Corporation, Tokyo, Japan) was assessed for inflammatory response. Trans epidermal water loss (TEWL, measured using VAPO SCAN, ASCH JAPAN Co., Ltd., Tokyo, Japan), which reflects the cutaneous barrier function, and itching behavior (observed for 60 min via video recording) were monitored immediately before the final imiquimod treatment. After the mice were euthanized under isoflurane inhalation anesthesia, back skin tissue and auricular lymph nodes (LNs) were sampled for further evaluation.

### 5.3. Histopathological Evaluation of Back Skin

The back skin samples were fixed in 10% formalin solution, embedded in paraffin wax, sectioned to a thickness of 5 μm, and stained with hematoxylin and eosin. A histopathological evaluation of parakeratosis in the epidermis, hyperplasia in the keratinized layer, inflammatory cell infiltration in the epidermis, hyperplasia in the non-keratinized layer, and inflammatory cell infiltration in the dermis was conducted in a blinded manner by a pathologist using the following grading system: 0, within normal limits; 1, mild; 2, moderate; and 3, severe.

### 5.4. Immunological Analysis of LNs

Single-cell suspensions of the LNs were used for flow cytometry and cytokine secretion assays. One million cells, treated with 1 µg of mouse Fc Block (Miltenyi Biotec K.K., Tokyo, Japan), were incubated with FITC-conjugated anti-mouse CD3, PE-conjugated anti-mouse CD4, FITC-conjugated anti-mouse CD11c, and PE-conjugated anti-mouse MHC class II antibodies (Miltenyi Biotec K.K.). Ten thousand events were analyzed for cell surface marker expression using an EC800 flow cytometer (Sony Imaging Products & Solutions Inc., Tokyo, Japan). One million cells were also incubated with Dynabeads Mouse T-Activator CD3/CD28 (DB11452, Thermo Fisher Scientific, Inc., Yokohama, Kanagawa, Japan) for 24 h. IL-17 and TNF-α concentrations in the supernatant were evaluated using an enzyme-linked immunosorbent assay (ELISA) (DY421 and DY410, R&D Systems, Minneapolis, MN, USA). The optical density at 450 nm was measured using a microplate reader (iMark Microplate Reader, Bio-Rad Laboratories, Inc., Hercules, CA, USA).

### 5.5. Cytokine Gene Expression in Back Skin Tissue

Back skin tissue was homogenized using a bead-beater homogenizer (μT-12, TAITEC CORPORATION, Saitama, Japan), and total RNA was extracted using the NucleoSpin^®^ RNA kit (TaKaRa Bio Inc., Kusatsu, Shiga, Japan). The extracted total RNA (500 ng) was reverse-transcribed using the PrimeScript™ RT Master Mix (TaKaRa Bio Inc.). The expression of β-actin (Forward: CATCCGTAAAGACCTCTATGCCAAC, Reverse: ATGGAGCCACCGATCCACA) and IL-17 (Forward: GAAGGCCCTCAGACTACCTCAA, Reverse: TCATGTGGTGGTCCAGCTTTC) was assessed using specific primers (TaKaRa Bio Inc.), PowerUp™ SYBR™ Green Master Mix (Thermo Fisher Scientific, Inc.), and a qPCR system (CFX Duet Real-Time PCR System, Bio-Rad Laboratories, Inc.). IL-17 gene expression was normalized to that of β-actin.

### 5.6. Statistical Analysis

All data are expressed as the mean ± standard error of the mean (SEM). An unpaired *t*-test with Welch’s correction was used to determine the significant differences between the values of the two groups. Statistical significance was set at 5% probability levels. Data were analyzed using GraphPad Prism 10 (GraphPad Software, San Diego, CA, USA).

## Figures and Tables

**Table 1:** Histological evaluation of back skin in a mouse psoriasis model. A histological score (0, within normal limits; 1, mild; 2, moderate; and 3, severe) was given for each observation. The results are expressed as the mean ± SEM. *n* = 8 per group. * *p* < 0.05, ** *p* < 0.01 (unpaired *t*-test) vs. the imiquimod control group.

Group	Imiquimod control	DON
Parakeratosis in epidermis	0.00 ± 0.00	1.00 ± 0.00
Hyperplasia in keratinized layer	2.88 ± 0.13	3.00 ± 0.00
Inflammatory cell infiltration in epidermis	0.40 ± 0.16	1.40 ± 0.16 *
Hyperplasia in non-keratinized layer	1.13 ± 0.13	2.00 ± 0.00 **
Inflammatory cell infiltration in dermis	2.00 ± 0.33	3.00 ± 0.00 *

## Data Availability

The original contributions presented in this study are included in this article. Further inquiries can be directed to the corresponding author.

## Abbreviations

The following abbreviations are used in this manuscript:

DON	deoxynivalenol
TEWL	transepidermal water loss
Th2	type 2 helper T cell
IL	interleukin
LNs	auricular lymph nodes
ELISA	enzyme-linked immunosorbent assay
SEM	standard error of the mean
